# Antioxidative CXXC Peptide Motif From Mesencephalic Astrocyte-Derived Neurotrophic Factor Antagonizes Programmed Cell Death

**DOI:** 10.3389/fcell.2018.00106

**Published:** 2018-09-04

**Authors:** Valentina Božok, Li-ying Yu, Jaan Palgi, Urmas Arumäe

**Affiliations:** ^1^Division of Gene Technology, Department of Chemistry and Biotechnology, Tallinn University of Technology, Tallinn, Estonia; ^2^Institute of Biotechnology, University of Helsinki, Helsinki, Finland

**Keywords:** CXXC motif, apoptosis, Fas, reactive oxygen species, MANF, dopaminergic neurons

## Abstract

Mesencephalic astrocyte-derived neurotrophic factor (MANF) is a potent survival-promoting protein with neurorestorative effect for neurodegenerative diseases. Its mechanism of action, albeit poorly known, depends strongly on the CXXC motif (CKGC). Here we studied the survival-promoting properties of the CKGC tetrapeptide from MANF. In the Jurkat T lymphocytic cell line, CKGC potently inhibits death receptor Fas-induced apoptosis and mildly counteracts mitochondrial apoptosis and necroptosis. The peptide with serines instead of cysteines (SKGS) has no survival-promoting activity. The cytoprotective efficiency of the peptide against Fas-induced apoptosis is significantly improved by reduction of its cysteines by dithiotreitol, suggesting that it protects the cells via cysteine thiol groups, partially as an antioxidant. CKGC neutralizes the reactive oxygen species, maintains the mitochondrial membrane potential and prevents activation of the effector caspases in the Jurkat cells with activated Fas. The peptide does not require intracellular administration, as it is endocytosed and resides mainly in the Golgi. Finally, the peptide also potently promotes survival of cultured primary dopaminergic neurons.

## Introduction

Programmed cell death is an essential process to remove unwanted or damaged cells, both in physiological and pathological situations. Cells can die via several death programs, including mitochondrial or death receptor-mediated apoptosis (Lavrik, 2014). Apoptosis is irreversible when caspases, the proteases that cause cellular demise, are activated above certain threshold. In mitochondrial apoptosis, caspases are activated by the proteins, such as cytochrome c, Smac/Diablo, etc., that are released from the mitochondria to cytosol (Estaquier et al., 2012; Tait and Green, 2013). In the death receptor mediated apoptosis, caspases are activated at the cytoplasmic domains of the ligated death receptors, such as Fas, TNF-α, and TRAIL (Lavrik and Krammer, 2012; Wajant, 2014). Cells can also die via non-apoptotic programs, such as necroptosis that can be activated mostly in pathological cases, although recently its involvement in physiological cell death has also been shown (Linkermann and Green, 2014; Grootjans et al., 2017).

Reactive oxygen species (ROS) are various highly reactive derivatives of oxygen, the most important ones being superoxide and hydrogen peroxide. Cellular ROS are mainly generated in the mitochondrial respiratory chain, but also by other cellular systems, such as cytochrome P450 enzymes (Zorov et al., 2014). Normally the cellular antioxidative mechanisms of defense, including enzymatic (superoxide dismutase, catalase, glutathione peroxidases, and thioredoxins) and non-enzymatic (ascorbate, glutathione, flavonoids, etc.) systems, maintain ROS at physiological levels (He et al., 2017). However, overproduction of ROS during cellular stress can overwhelm the antioxidant systems and, if not alleviated, lead to oxidative stress, oxidative damage of the cellular constituents and cell death. ROS are excessively generated and actively participate in all cell death programs (Kim et al., 2010; Cheng et al., 2014; Gonzalez-Flores et al., 2014; Zhang et al., 2015; Redza-Dutordoir and Averill-Bates, 2016). Importantly, elevated ROS are also associated with multiple pathologies, including neurodegenerative diseases, cancer, diabetes, etc., and prevention of ROS could be a promising therapeutic approach (Bachnoff et al., 2011; Mailloux et al., 2013; McManus et al., 2014).

Mesencephalic astrocyte-derived neurotrophic factor (MANF) and cerebral dopamine neurotrophic factor (CDNF) constitute an evolutionarily conserved family of genes that are conserved throughout the animal kingdom, the vertebrates having both orthologous genes, while the invertebrates have only single ancestral orthologue (Lindholm et al., 2007; Lindstrom et al., 2013; Voutilainen et al., 2015). Both proteins potently promote cell survival. These proteins are currently the most promising treatment candidates in several neuropathological conditions, but also in diabetes (Voutilainen et al., 2015; Lindholm et al., 2016; Lindahl et al., 2017). Most importantly, MANF and especially CDNF are neurorestorative in the animal models of Parkinson’s disease. Indeed, when applied to the animals with severe chemically induced Parkinsonian symptoms, these factors significantly improve the neurological scores and reduce histological brain damages (Lindholm et al., 2007; Airavaara et al., 2009; Voutilainen et al., 2009, 2011; Huotarinen et al., 2018). MANF is also neurorestorative in the rat model of cerebral stroke (Matlik et al., 2018). The cytoprotective mechanisms of MANF and CDNF are currently poorly understood. Both can protect the cells as intracellular endoplasmic reticulum proteins but at least in the *in vivo* experiments, also as secreted factors. However, the molecular details for either mode of action are not yet clear (Voutilainen et al., 2015).

Structurally MANF consists of N- and C-terminal domains that are connected by flexible linker (Parkash et al., 2009; Hellman et al., 2011). Two short motifs have been identified on the C-terminal domain of MANF that are functionally important for its cytoprotective activity: C-terminal KDEL-like endoplasmic reticulum retention motif (RTDL) and the CXXC motif (CKGC) (Matlik et al., 2015). The CXXC motif is evolutionarily conserved, being always found in the same conserved position in the structure of the MANF and CDNF, i.e., in the loop region between two alpha-helices in the C-terminal domains of the proteins (Parkash et al., 2009; Hellman et al., 2011). It seems to be a critical functional motif of the protein. Indeed, overexpressed MANF has anti-apoptotic effect on the sympathetic and sensory neurons *in vitro*, and infused MANF protein protects the cerebral cortex in rat model of cerebral ischemia (Airavaara et al., 2010; Hellman et al., 2011; Matlik et al., 2015). In contrast, MANF having the C-terminal cysteine of the CXXC motif replaced by serine (MANF-CKGS) is completely inactive in both models (Matlik et al., 2015). However, the exact role of the CXXC motif in the cytoprotective activity of MANF (and CDNF) is not yet clear.

Cysteine motifs, including CXXC, are common in the catalytic centers of the enzymes performing disulfide isomerization or redox reactions. In such motifs one of the cysteines usually initiates disulfide formation or reduction. This process generally leads to changes in the structure of the target proteins that in turn modulates their activity. Cysteine motifs can also reduce and thereby inactivate ROS, thus alleviating dangerous oxidative stress in the cells (Wouters et al., 2010). Not surprisingly, cysteine motifs are present in the proteins involved in oxidative stress, such as oxidoreductases and thioredoxins (Wouters et al., 2010; Shaw et al., 2014). However, the antioxidant activity is not the only function of the CXXC motifs. Some cysteine domains function also by binding metal ions. E.g., thioredoxin 2 has a zinc binding CXXC motif (Collet et al., 2003), and the CXXC motifs in the metal binding domains of ATP7B coordinate copper ions (Safaei et al., 2012). There could be still additional modes of action for the CXXC motif.

Here we set up to study the survival-promoting activity of MANF-derived CKGC peptide. We show that in Jurkat cells, the reduced non-cyclic CKGC peptide efficiently protects the cells against Fas-mediated apoptosis and this protection correlates with the reduction of the levels of ROS and the maintenance of mitochondrial membrane potential. Although in this study we focused on death receptor-dependent apoptosis, CKGC peptide has a wider range of activity being able to counteract also mitochondrial apoptosis and necroptosis. The peptide enters the cells, presumably by endocytosis, and retains mainly in the Golgi. The peptide also potently promotes survival of cultured primary dopaminergic neurons. Studying the activity of the CKGC peptide would potentially help to reveal new possibilities in discovery and delivery of drugs against neurodegenerative diseases, but also to find out the details about mechanisms of action of MANF/CDNF that are yet poorly understood.

## Materials and Methods

### Cell Culture

Jurkat cells (immortalized human T lymphocyte cell line) were maintained in RPMI-1640 medium with L-glutamine and sodium bicarbonate (Sigma-Aldrich, Irvine, United Kingdom) and HeLa cells in the Dulbecco’s modified Eagle’s medium with GlutaMAX (Life Technologies, Paisley, United Kingdom). Media were supplemented with 10% fetal bovine serum (FBS) and Penicillin (100 U/ml)/Streptomycin (100 μg/ml) (all from PAA Laboratories, Cölbe, Germany). Cell cultures were grown in humidified incubator at 37°C in 5% CO_2_ atmosphere until the cells reach 50–70% confluence. Dissociated cultures of embryonic day (E) 13 NMRI mouse midbrain floors were prepared as published (Yu and Arumae, 2008; Yu et al., 2008). All procedures for animal use were approved by the University of Helsinki Laboratory Animal Centre (Protocol number KEK11-020). Briefly: the neurons were grown on the poly-ornithine (Sigma-Aldrich) coated microisland areas scratched to the cell culture dishes, in DMEM/F12 medium (Gibco/Thermo Fisher Scientific, Rockford, IL, United States) containing N2 serum supplement (Gibco/Thermo Fisher Scientific), 33 mM D-glucose (Sigma-Aldrich), and L-glutamine (Gibco/Thermo Fisher Scientific) in the presence of the peptides at increasing concentrations. Glial cell line-derived neurotrophic factor (GDNF) (PeproTech, Ltd., London, United Kingdom) at 100 ng/ml was included as a positive survival-promoting control for dopaminergic neurons and cultures without any added survival-promoting factor served as a negative control showing the extent of cell death. After 5 days *in vitro* the cultures were stained with antibodies to tyrosine hydroxylase (CHEMICON, Temecula, CA, United States) to reveal dopaminergic neurons among other neuronal types in these mixed cultures. All immunopositive neurons were counted manually under the microscope and expressed as percent of GDNF-maintained neurons. The experiments were repeated on seven independent cultures.

### Peptides

The following peptides were supplied by CASLO (Lyngby, Denmark): CKGC (Ac-Cys-Lys-Gly-Cys-NH2), dCKGC (Ac-dCys-dLys-dGly-dCys-NH_2_), GCCK (Ac-Gly-Cys-Cys -Lys-NH_2_), GCCG (Ac-Gly-Cys-Cys-Gly-NH_2_), KCCG (Ac-Lys-Cys-Cys-Gly-NH_2_), CGCK (Ac-Cys-Gly-Cys-Lys-NH_2_), SKGS (Ac-Ser-Lys-Gly-Ser-NH_2_), FITC-CKGC (FITC-Cys-Lys-Gly-Cys-NH_2_), and FITC-SKGS (FITC-Ser-Lys-Gly-Ser-NH_2_). All peptides used in this study were acetylated (Ac) at N-terminus and amidated (NH_2_) at C-terminus by manufacturer, except FITC-CKGC and FITC-SKGS, that had FITC at N-terminus and were amidated at C-terminus. Modification of the termini is required, as peptides with open cysteine termini (CKGC and dCKGC) caused severe cell damage, while non-modified SKGS was not harmful (not shown). In addition, acetylation and amidation of termini are known to increase the stability and cell membrane permeability of the peptides.

Reduction of the cysteines was performed by preincubation of the peptides, including the control peptide SKGS, with 4.5 μM dithiothreitol (DTT) (Roche, Mannheim, Germany) in Milli-Q water for 30 min at 37°C. Peptides were added to the culture medium with or without DTT pretreatment depending on the experimental design.

### Induction of Apoptosis and Necroptosis

In all death and survival assays, the apoptosis-inducing (etoposide and anti-Fas antibody) and anti-apoptotic (peptides and caspase inhibitor) agents were added at the same time. Death receptor-dependent apoptosis was induced by 5 ng/ml agonistic anti-human Fas monoclonal antibody, clone CH11 (Millipore, Temecula, CA, United States; catalog # 05-201). Five μM etoposide (Sigma-Aldrich, Schnelldorf, Germany) was used to initiate mitochondrial apoptosis. Necroptosis was induced by anti-Fas antibody CH11 (5 ng/ml) in the presence of 5 μM pan-caspase inhibitor Q-VD-OPh (Sigma-Aldrich, Schnelldorf, Germany). Temporally it takes approximately up to 6, 10 h, and 1–2 days for the first morphological signs of cell death to appear in death receptor-dependent apoptosis, mitochondrial apoptosis and necroptosis, respectively. The duration of cell incubation with toxins and counteracting agents was chosen individually for each experiment.

### Assays for Cell Viability and Death

Jurkat cells in the exponential phase of growth were seeded on the white-walled 96-well microplate wells with transparent bottom (Greiner Bio-One, Frickenhausen, Germany), 2 × 10^4^ cells per well in 100 μl of medium. Appropriate toxin and tetrapeptide were added to the cells at the same time and maintained for indicated time periods at 37°C, 5% CO_2_. As a positive control, pan-caspase inhibitor Q-VD-OPh (5 μM) was added to the toxin-treated cells. The positive control treatment for necroptosis assay included 5 ng/ml of anti-Fas antibody, 5 μM of Q-VD-OPh and 20 μM of necrostatin-1 (Nec-1) (Santa Cruz Biotechnology, Santa Cruz, CA, United States) as a specific inhibitor of necroptosis. Nec-1 was preloaded for 1 h before adding the rest of the agents. As the negative controls, toxin alone or toxin plus inactive tetrapeptide (SKGS) were used.

Cell viability was estimated by measuring the levels of intracellular ATP using CellTiter-Glo reagent (Promega, Madison, WI, United States). CellTiter-Glo was added with the ratio 1:1 (100 μl per well) at the end of each treatment assay. The plate was manually shaked for two minutes to facilitate component mixing and cell lysis. After 15 min of incubation at room temperature (RT) in the dark, the luminescence was measured with Tecan GENios Pro microplate reader (Tecan Group, Switzerland).

In some experiments the extent of cell death was measured in addition to the cell viability. To measure the amount of dead cells, 250 nM SYTOX Green nucleic acid stain (Life Technologies, Eugene, OR, United States) that detects the cells with compromised plasma membrane was added to the cells without washing. The cells were then incubated for 15 min at RT in the dark and the fluorescence was measured with Tecan GENios Pro microplate reader using 492 nm excitation and 535 nm emission filters. Cell death assay was performed prior to measurement of ATP levels from the same wells.

To measure the activity of caspase-3/7 the cells were incubated with CH11 antibody and DTT-pretreated peptide (CKGC, dCKGC, or SKGS) or pan-caspase inhibitor Q-VD-OPh for 4 h at 37°C, 5% CO_2_. Caspase-Glo 3/7 reagent (Promega, Madison, WI, United States) was added with ratio 1:1 (100 μl per well) and incubated for 30 min at RT in dark before the luminescence was measured with Tecan GENios Pro microplate reader. Caspase-8 activity was measured by similar procedure, except that the cells were incubated with peptides for 3 h and then for 1 h with Caspase-Glo 8 reagent premixed with MG-132 protease inhibitor (Promega).

### Detection of Reactive Oxygen Species and Intact Mitochondria

To study the antioxidative properties of the CXXC tetrapeptides in cell-free conditions, 50 μl of Stable Peroxide Buffer from SuperSignal West Femto Maximum Sensitivity Substrate kit (Thermo Fisher Scientific, Rockford, IL, United States) was incubated with 500 μM of dCKGC or SKGS peptide pretreated with 4.5 μM DTT, or equal amount of Milli-Q water. As a control, DTT was added to the peroxide buffer. The solutions were maintained for 10 min at RT in white-walled 96-well plate to allow the peptides to react with H_2_O_2_. Horseradish peroxidase-conjugated antibody (Thermo Fisher Scientific, Rockford, IL, United States; catalog # 32430) was mixed with Luminol Enhancer Solution containing Horseradish Peroxidase Substrate and 50 μl of the mix was added to each well. Final dilution of the antibody was 1:25000. The plate was incubated for 5 min at RT in dark and the luminescence measured by Tecan GENios Pro microplate reader. 100, 75, 50, 25, and 0% of Stable Peroxide Buffer Milli-Q water solutions were used as a reference for the calculation of the amount of neutralized hydrogen peroxide.

To measure the level of intracellular ROS, Jurkat cells were plated onto 96-well white-walled microplate wells with transparent bottom at 2 × 10^4^ cells per well. Serum free RPMI-1640 medium was used because serum seems to impede the detection of fluorescent signal in this assay. The cells were treated with 5 ng/ml of anti-Fas antibody CH11 to induce extrinsic apoptosis. CKGC or SKGS peptide were added at the concentration 100 or 500 μM. For the positive control, cells were treated with the combination of CH11 and 5 μM of pan-caspase inhibitor Q-VD-OPh. The plate was incubated at 37°C and 5% CO_2_ for 6 h, then 5 μM of CM-H2 DCFDA (general ROS indicator) or 5 μM of MitoSOX Red Mitochondrial Superoxide Indicator (both from Thermo Fisher Scientific, Rockford, IL, United States) was added and the plate was kept for 10 min at RT in dark. In order to detect the integrity of mitochondria, the cells were plated similarly and treated with CH11 antibody and/or the peptide (CKGC or SKGS) preincubated with DTT. Equal amount of DTT was added to the controls. After 18 h of incubation at 37°C and 5% CO_2_ 100 nM of MitoTracker Red CMXRos fluorescent dye (Thermo Fisher Scientific, Rockford, IL, United States) was added and incubated for 10 min at RT in dark. The images of stained cells were taken with Zeiss Axiovert 200 M fluorescence microscope (Zeiss, Germany). The fluorescence of CM-H2 DCFDA was measured by Tecan GENios Pro microplate reader using 492/535 nm excitation/emission filter.

### Transfection of HeLa Cells

HeLa cells in the exponential phase of growth were seeded onto glass coverslips in 48-well plate wells (Greiner Bio-One, Frickenhausen, Germany), 1.5 × 10^4^ cells per well in 200 μl of culture medium. Next day the cells were transfected using Lipofectamine 2000 transfection reagent (Invitrogen, Carlsbad, CA, United States). Plasmid DNA/lipofectamine complex was prepared in DMEM medium (PAA Laboratories, Cölbe, Germany) without FBS, Penicillin-Streptomycin and phenol red and applied according to the manufacturer’s instructions (0.25 μg of plasmid DNA and 0.8 μl of lipofectamine reagent per well). Culture medium was changed at 4 h after transfection to avoid the toxic influence of lipofectamine reagent. pDsRed2-Mito plasmid was obtained from Clontech (Mountain View, CA, United States) and mCherry-TGNP-N-10 was a gift from Michael Davidson (Addgene plasmid # 55145).

### Analysis of Cell Penetration by CKGC Peptide and Its Intracellular Localization

One day after transfection the HeLa cells were treated with 100 μM of DTT-pretreated CKGC or SKGS peptide, N-terminally conjugated with fluorescein isothiocyanate (FITC). As CKGC peptide is prone to aggregation in the pH slightly higher than neutral, the brightly fluorescent aggregates were removed as follows. Conditioned medium from the cells was transferred to 1.5 ml tube, DTT-pretreated FITC-CKGC or FITC-SKGS was added, the solution was mixed by vortexing, briefly centrifuged and returned to the cells. The action was performed as quickly as possible not to allow the cells to dry. The cells were then incubated for 1 h at 37°C and 5% CO_2_, washed with phosphate-buffered saline (PBS) for three times and fixed with 4% paraformaldehyde (PFA) (Sigma-Aldrich, Steinheim, Germany) for 15 min at RT. Fixed cells were washed twice with PBS and once with Milli-Q water and mounted in ProLong Diamond Antifade Mountant, containing 4′,6-diamidino-2-phenylindole (DAPI) (Thermo Fisher Scientific, Rockford, IL, United States) to reveal cell nuclei. Images were taken using Zeiss LSM 510 META confocal microscope (Zeiss, Germany). Co-localization analysis was made using Imaris 6.4.2 software (Bitplane, Switzerland). Co-localization of signals from two channels on confocal microscopy image were interpreted by Pearson’s correlation coefficient (PCC). PCC = 1 if the intensity of all pixels from two channels matches perfectly and PCC = -1 means perfect inverse correlation of two channels (Dunn et al., 2011). Jurkat cells were incubated with 100 μM of FITC-CKGC or FITC-SKGS peptide in RPMI-1640 medium supplemented with FBS for 1 h at 37°C and 5% CO_2_. Then the cells were pelleted by centrifugation, washed twice with RPMI-1640 without FBS and plated onto glass coverslips in the 48-well plate wells. In the absence of serum, the cells mildly attached during about 15 min. The medium was then carefully replaced with 4% PFA solution. Fixed cells were washed and mounted as described above. The specimens were observed using Zeiss Olympus BX61 epifluorescence microscope (Zeiss, Germany) and the images were merged using Adobe Photoshop CC 2015.

### Immunocytochemistry

HeLa cells grown on coverslips in the 48-well plate were fixed with 4% PFA, washed twice with PBS, permeabilized with PBS containing 0.5% Triton X-100 (Amresco, Solon, OH, United States) and 50 mM NaCl (Sigma-Aldrich, Steinheim, Germany) for 10 min at RT and washed three times with PBS. Non-specific sites were blocked using 2% bovine serum albumin (BSA) (Sigma-Aldrich, Schnelldorf, Germany) solution in PBS for 1 h at RT. Then blocking solution was substituted to 0.2% BSA in PBS with mouse anti-GM130 antibody, labeling cis-Golgi compartment (BD Biosciences, San Jose, CA, United States; catalog # 610823) diluted at 1:500. The cells were kept overnight at 4°C, then washed three times with 0.05% Tween 20 (Sigma-Aldrich, Steinheim, Germany) solution in PBS. Alexa Fluor 488 goat-anti mouse IgG antibody (Invitrogen, Eugene, OR, United States; catalog # A11017) in 0.2% BSA/PBS (1:2000) was added and kept for 1 h at RT in dark. After three washes with PBS/Tween 20, coverslips were rinsed in Milli-Q water, slightly drained by filter paper tissue and mounted in ProLong Diamond Antifade Mountant with DAPI. Images were taken using Zeiss LSM 510 META confocal microscope. The Jurkat cells, placed onto glass coverslips as described above, were stained with antibodies in the same way, observed with Zeiss Olympus BX61 epifluorescence microscope (Zeiss, Germany) and the images were merged with Adobe Photoshop CC 2015.

### Statistical Analysis

All quantitative results are reported as the means ± SEM of at least three separate experiments on the independent cultures (*n* ≥ 3), each experimental point always being done in two or three parallels. The means were statistically compared by one-way ANOVA test with Dunnett’s post-test, or with unpaired *t*-test, using the program GraphPad InStat 3.10 (La Jolla, CA, United States). The data groups used for normalization were always excluded from statistical comparisons.

## Results

### CKGC Peptide Weakly Protects Jurkat Cells Against Several Programmed Cell Death Modes

**FIGURE 1:**
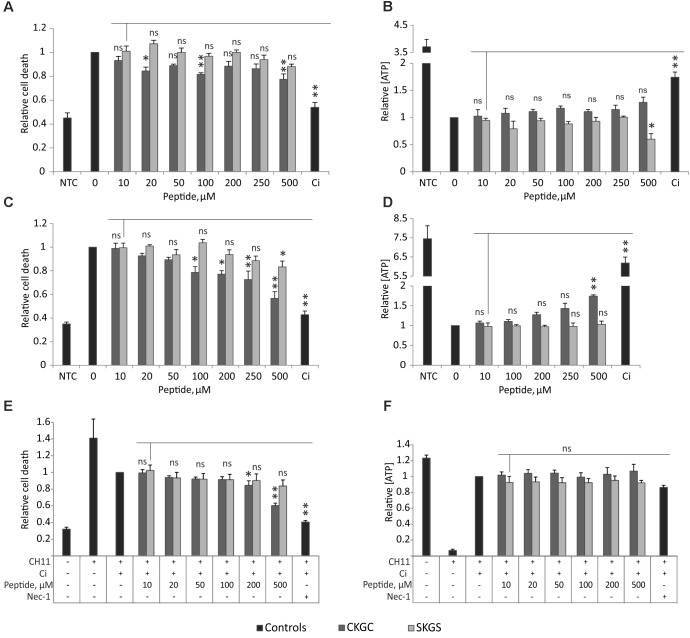
CKGC peptide counteracts different types of programmed cell death. CKGC peptide slightly inhibits cell death during mitochondrial apoptosis **(A,B)**, death receptor-induced apoptosis **(C,D)** and necroptosis **(E,F)**. Jurkat cells were co-treated with cells death inducing agent and CKGC peptide or the control SKGS peptide at indicated concentrations. Five μM etoposide was used for the induction of mitochondrial apoptosis **(A,B)**, 5 ng/ml Fas-activating antibody CH11 – for death receptor-induced apoptosis **(C,D)** and the combination of 5 ng/ml CH11 antibody and 5 μM pan-caspase inhibitor Q-VD-OPh (Ci) – for necroptosis **(E,F)**. In the control cells, apoptosis was blocked by 5 μM pan-caspase inhibitor Q-VD-OPh (Ci) and necroptosis with 20 μM necrostatin-1 (nec-1). NTC indicates the non-treated cells. After 16 **(A–D)** or 72 h **(E,F)** of incubation the cells were stained with dead cell-detecting dye SYTOX Green **(A,C,E)**, or the concentration of ATP was measured **(B,D,F)**. All values were normalized to those of the toxin-only-treated controls. Shown are the means ± SEM, *n* = 3 (apoptosis), *n* = 4 (necroptosis). Statistical significance between the groups was calculated by one-way ANOVA with Dunnett’s post-test comparing the experimental groups to the 10 μM SKGS treated group (indicated by the vertical line); the statistical analysis was performed separately for CKGC and SKGS peptides. ^∗^*p* < 0.05; ^∗∗^*p* < 0.01; ns, not significant.

We hypothesized that the CKGC tetrapeptide of MANF could have cytoprotective properties of its own. We tested this hypothesis on Jurkat T-cell line, as these cells can be induced to die via different cell death modes (Valavanis et al., 2001; Karpinich et al., 2006; Li and Shively, 2013; Sosna et al., 2014). We killed Jurkat cells via different ways: classical apoptosis induced with etoposide, death receptor-dependent apoptosis induced by agonistic antibody CH11 to Fas receptor, and necroptosis achieved by activation of Fas receptor for a long time (72 h) in the presence of pan-caspase inhibitor Q-VD-Oph. We measured the amount of dead cells by SYTOX Green staining, and the amount of actively metabolizing cells by the level of ATP. CKGC peptide added to the culture medium was able to rescue Jurkat cells from all three types of programmed cell death (**Figure 1**). However, this rescue was significant only at higher concentrations (500 μM) and did not reach the death-blocking efficiency of caspase inhibitor. The control peptide SKGS did not have a significant survival-promoting effect. Although the CKGC peptide reduced the number of cells with compromised plasma membranes, as detected by the SYTOX Green dye, it did not manage to prevent the drop in the level of ATP, an important indicator of cell viability. Thus, the peptide only postponed the programmed cell death.

### Reduction of Cysteines Improves Cytoprotective Effect of the CKGC Peptide Against Fas-Induced Apoptosis

**FIGURE 2:**
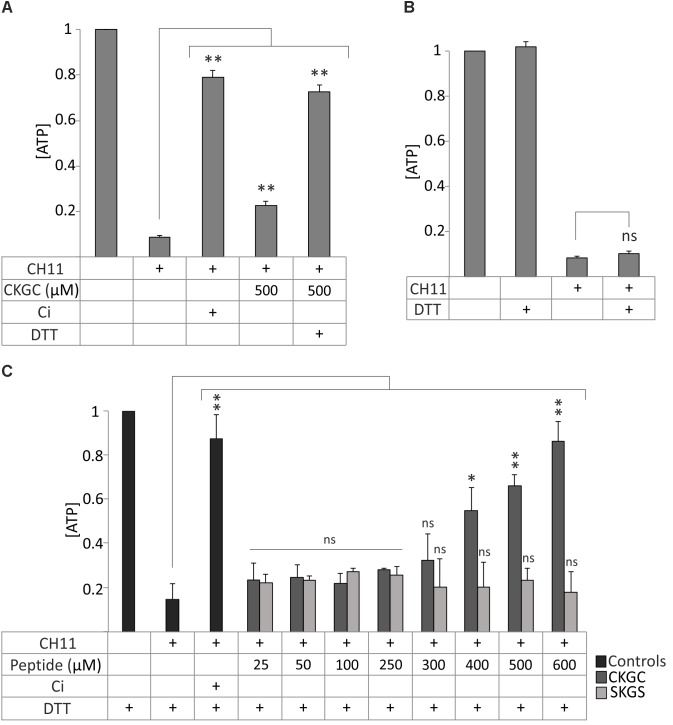
Reduction of CKGC tetrapeptide improves its cytoprotective properties against Fas-induced apoptosis. **(A)** Jurkat cells were treated with Fas-activating antibody CH11 and with 500 μM CKGC peptide with or without DTT pretreatment. The control contains 5 μM of pan-caspase inhibitor Q-VD-OPh (Ci). The levels of ATP were measured after 18 h and normalized to those of non-treated cells. Reduction of the peptide significantly improves its cytoprotective ability. **(B)** Jurkat cells were incubated with 0.2 μM DTT, the final concentration of DTT in **(A)** or equal amount of solvent, and the levels of ATP were measured after 18 h. The improved cytoprotective effect of the DTT-reduced CKGC peptide in **(A)** was not caused by DTT itself. **(C)** Jurkat cells were treated with Fas-activating antibody CH11 and the DTT-pretreated CKGC or SKGS control peptides at indicated concentrations. Five μM of caspase inhibitor (Ci) was added to the control cells. DTT-treated CKGC peptide dose-dependently protected the cells against activated Fas, whereas DTT-treated SKGS peptide was without effect. All values were normalized to cells exposed to minimal treatment. Shown are the means ± SEM, *n* = 3. Statistical significance between the groups in **(A,C)** was calculated by one-way ANOVA with Dunnett’s post-test comparing the experimental groups to the CH11-only treated group (indicated by the long vertical lines), and the groups in **(B)** were compared by Student’s *t*-test; ^∗^*p* < 0.05; ^∗∗^*p* < 0.01; ns, not significant.

We next attempted to improve the cytoprotective efficiency of the peptide. Applying additional portion of the peptide to the cells in the middle of incubation did not improve the result (not shown). Thus, its low cytoprotective efficiency was not caused by rapid degradation. We then paid attention to the pair of cysteines in the CXXC motif that could be responsible for the functionality of the peptide. We realized that CKGC peptide might be active only when the cysteines are in the reduced status. The thiol groups of the cysteines are highly reactive (McBean et al., 2015) and could be easily oxidized after solubilization of the lyophilized peptide, thereby reducing its activity. Another possible problem could be polymerization of the CKGC peptide by oxidation of its cysteines. To prevent these potentially inactivating reactions we reduced the peptide by disulfide reducing agent dithiothreitol (DTT). Pretreatment of the peptide with DTT for 30 min at 37°C drastically improved its cytoprotective ability against Fas-induced apoptosis, as revealed by the maintenance of the ATP levels. Thus, the DTT-CKGC treated Jurkat cells showed about 70% higher viability compared to the cells treated with non-reduced peptide (**Figure 2A**). DTT itself did not influence ATP levels in the control cells (**Figure 2B**), even though it has a redox system composed of two thiol groups similarly to the CKGC peptide. However, being a well-known reducing agent DTT might still affect the redox status of the cells. Therefore, in the following experiments DTT was always added to the control wells. As the reduced peptide most potently antagonized Fas-induced apoptosis and much less the mitochondrial apoptosis and necroptosis (not shown), we performed the following experiments on this model.

The effect of DTT pretreated CKGC peptide against Fas-induced apoptosis was dose-dependent, starting from 400 μM and continuing up to 600 μM, the highest concentration tested (**Figure 2C**). At 600 μM, the cytoprotective effect still did not reach the plateau, although at this point about 89 ± 7% (SEM) of cells were rescued that matches the effect of caspase inhibitor (90 ± 9%, SEM) (**Figure 2C**). The control peptide SKGS has no effect with (**Figure 2C**) or without (**Figure 1D**) DTT pretreatment. These experiments show the reductive nature of active CKGC peptide and functional importance of its cysteine pair.

### The Position of Cysteines and Lysine in the CKGC Tetrapeptide Affects Its Cytoprotective Function

**FIGURE 3:**
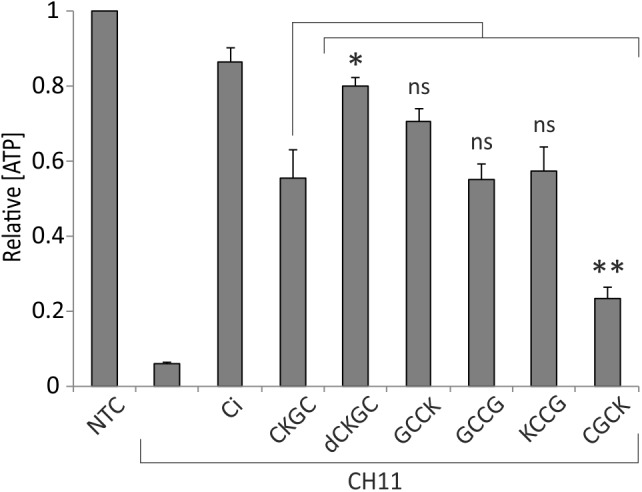
Composition of CXXC motif peptide influences its cytoprotective function. Jurkat cells were incubated for 18 h with Fas-activating antibody CH11 (5 ng/ml) and the indicated peptides at 500 μM. dCKGC denotes the enantiomer of the peptide composed of D-amino acids. All peptides were pretreated with 4.5 μM DTT, and DTT was added to the wells without peptides at 0.2 μM (its final concentration in the peptide-treated cells). Five μM pan-caspase inhibitor Q-VD-OPh (Ci) was added to the control cells. The levels of ATP were measured after 18 h and normalized to those of the non-treated cells (NTC). Note that the CXXC and XCCX peptides are more potent anti-apoptotic agents than CXCX. The position of lysine as well as type of amino acid isomers also play role in cytoprotective ability of the peptide. Shown are the means ± SEM, *n* = 3. Statistical significance between the groups was calculated by one-way ANOVA with Dunnett’s post-test comparing the experimental groups to the CH11+CKGC group (indicated by the long vertical line); ^∗^*p* < 0.05; ^∗∗^*p* < 0.01; ns, not significant.

The absence of cytoprotective effects of the control peptide SKGS suggests that CKGC peptide rescues the cells via its cysteine residues. Also, the CKGC peptide composed of D-isomeric amino acids (dCKGC) efficiently protected the cells against Fas-induced apoptosis (**Figure 3**), further suggesting that the protection occurs via cysteine residues rather than via cysteine-independent interactions with other cellular molecules. However, the order and positions of amino acids of CKGC tetrapeptide may also influence its function. To figure this out we treated Jurkat cells with Fas receptor activating antibody CH11 and the following peptides: GCCK, GCCG, KCCG, and CGCK. The results presented in **Figure 3** show that GCCK, GCCG, and KCCG peptides protected the cells against activated Fas with similar efficiency as the original CKGC peptide, whereas the effect of CGCK was significantly smaller. Thus, not only the presence of two cysteines but also the number of interjacent amino acids and the position of lysine are the important parameters for the cytoprotective ability of the peptide. dCKGC was more effective than the original L-enantiomer of the peptide, presumably because of its resistance to intracellular proteases.

### CKGC Peptide Antagonizes Fas-Induced Rise of Reactive Oxygen Species in Jurkat Cells

**FIGURE 4:**
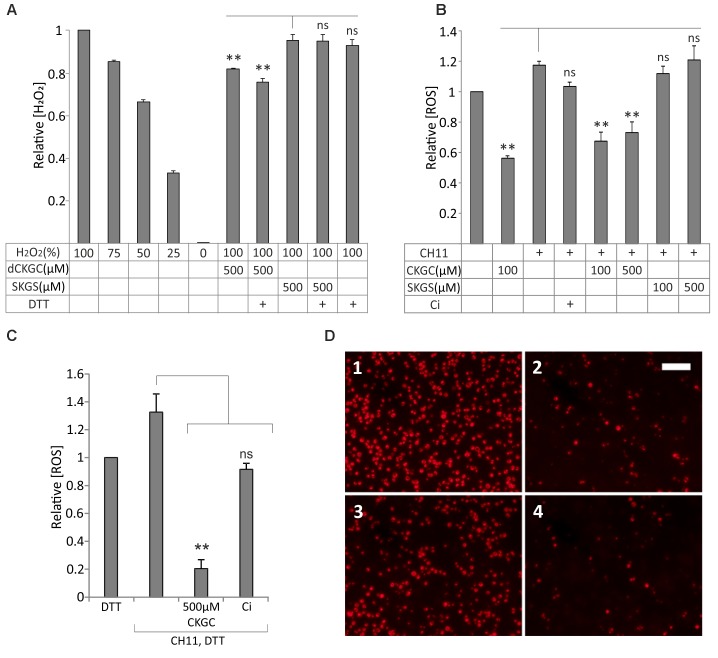
CKGC peptide inactivates reactive oxygen species (ROS) and preserves mitochondrial membrane potential. **(A)** dCKGC peptide neutralizes H_2_O_2_ in cell-free conditions. dCKGC or SKGS peptides were preincubated with 4.5 μM DTT or equal amount of solvent and their ability to neutralize H_2_O_2_ was determined as specified in the Methods. All values were normalized to 100% H_2_O_2_ controls. **(B,C)** Reduced CKGC peptide lowered the levels of ROS in the Fas-treated Jurkat cells. The cells were treated with Fas-activating antibody CH11 (5 ng/ml) and the DTT-treated **(C)** or non-treated **(B)** CKGC or SKGS peptide at indicated concentrations. In the control cells, apoptosis was blocked by 5 μM pan-caspase inhibitor Q-VD-OPh (Ci). The cultures were incubated for 6 h in the absence of serum and stained with fluorescent ROS indicator CM-H2 DCFDA. All values were normalized to those of CH11 not treated cells. Shown are the means ± SEM, *n* = 3. Statistical significance between the groups was calculated by one-way ANOVA with Dunnett’s post-test comparing the experimental groups to the SKGS-treated controls [**(A)** indicated by vertical line] and the CH11-only-treated controls [**(B,C)** indicated by vertical lines]. ^∗∗^*p* < 0.01; ns, not significant. **(D)** Jurkat cells were incubated with anti-Fas agonistic antibody CH11 and 500 μM DTT-treated CKGC or SKGS peptide for 18 h and stained with MitoTracker Red CMXRos fluorescent dye that is activated in normally respiring mitochondria. 1: DTT; 2: CH11+DTT; 3: CH11+CKGC; and 4: CH11+SKGS. Shown are the images made with fluorescent microscope. Scale bar 100 μm.

We hypothesized that CKGC peptide could protect the cells as an antioxidant, neutralizing ROS via its cysteine thiol groups. We tested this hypothesis first with H_2_O_2_ in cell-free conditions, using SuperSignal West Femto Maximum Sensitivity Substrate kit. In this experiment we used dCKGC considering that reaction with H_2_O_2_ does not depend on stereoisomerism of the peptide. 500 μM dCKGC or SKGS was incubated with H_2_O_2_-containing Stable Peroxide Buffer. The peptide with cysteines neutralized about 30% of the H_2_O_2_ molecules compared to the control peptide without cysteines (**Figure 4A**). DTT pretreatment of dCKGC raised the efficiency of the peptide by about 5%, while DTT alone was without effect.

We then studied whether the reduced CKGC peptide could neutralize ROS in the living Jurkat cells with activated Fas. Activation of Fas receptor by CH11 antibody raised the level of ROS in Jurkat cells, as shown by MitoSOX Red or CM-H2 DCFDA, the indicators of intracellular ROS (**Supplementary Figure 1**). As revealed by CM-H2 DCFDA, both 100 and 500 μM of the CKGC peptide potently reduced the Fas-induced levels of ROS, whereas the control SKGS peptide was without effect (**Figure 4B**). Jurkat cells have relatively high endogenous level of ROS that was also reduced by 100 μM of the CKGC peptide (**Figure 4B**). DTT pretreatment raised the ROS-neutralizing activity of CKGC almost four times (**Figure 4C**). Staining the Jurkat cells with Mitotracker Red CMXRos, the indicator of normal mitochondrial membrane potential, revealed the ability of DTT-treated CKGC to preserve mitochondrial functionality in CH11 antibody-treated cells, whereas SKGS was again without effect (**Figure 4D**).

### Reduced CKGC Peptide Inhibits Fas-Induced Caspase-8 and Caspase-3/7 Activation

**FIGURE 5:**
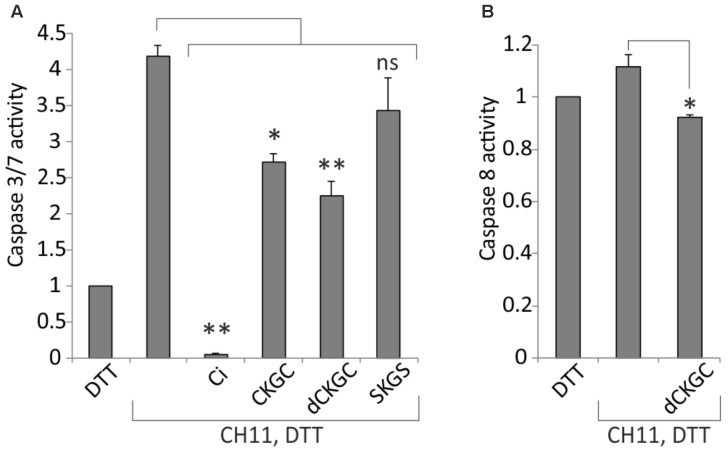
CKGC peptide decreases caspase-3/7 **(A)** and -8 **(B)** activity in Jurkat cells with activated Fas. Jurkat cells were treated for 4 h with Fas-activating antibody CH11 (5 ng/ml) and the indicated peptides at 500 μM, all reduced with 4.5 μM DTT. dCKGC denotes the enantiomer of the peptide composed of D-amino acids. In the control cells apoptosis was blocked by 5 μM of pan-caspase inhibitor Q-VD-OPh (Ci). Caspase activity was determined with Caspase-Glo 3/7 or -8 assays. The values were normalized to those of DTT-only treated cells. Shown are the means ± SEM, *n* = 3. Statistical significance between the groups was calculated by one-way ANOVA with Dunnett’s post-test comparing the experimental groups to the CH11-only treated group; ^∗^*p* < 0.05; ^∗∗^*p* < 0.01; ns, not significant.

Activation of caspases is a critical irreversible execution step in the apoptotic pathways. To study the influence of CKGC peptide on the caspase activation we co-treated Jurkat cells with Fas-activating antibody CH11 and the CKGC or control peptides and measured the level of activated caspase 8 and caspase 3/7, the initiator and effector caspases, respectively, in the death receptor pathway. As shown on **Figure 5A**, CKGC peptide, preincubated with DTT, decreased the activity of these effector caspases by 34.7 ± 4.4% (SEM) and dCKGC by 46.6 ± 4% (SEM) in CH11-treated Jurkat cells, compared to the CH11-only control. Caspase-8 activation was also significantly blocked by the peptide 17 ± 4% (SEM) (**Figure 5B**).

### CKGC Peptide Enters the Cells and Resides in the Golgi

**FIGURE 6:**
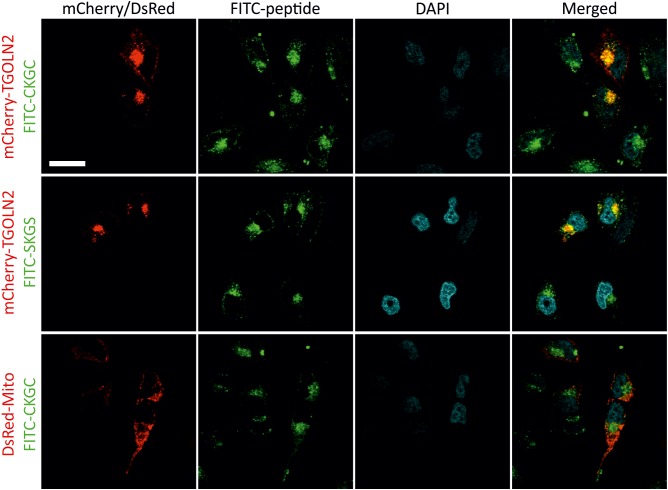
Intracellular localization of CKGC and SKGS peptides in HeLa cells. HeLa cells were transfected with mCherry-TGNP-N-10 plasmid encoding for the TGOLN2 marker of trans-Golgi network, or pDsRed2-Mito plasmid encoding for mitochondrially targeted DsRed. Transfected cells were treated with 100 μM of CKGC or SKGS peptides N-terminally conjugated with FITC for 1 h, fixed and examined by confocal microscopy. The peptides were preincubated with 4.5 μM DTT. Peptide-containing vesicles are mostly found in the perinuclear region where they co-localize with the marker for trans-Golgi network (FITC-CKGC: PCC = 0.55, *n* = 16; FITC-SKGS: PCC = 0.61, *n* = 5), but are absent in mitochondria (FITC-CKGC: PCC = –0.30, *n* = 11). The nuclei are revealed by DAPI. Scale bar, 20 μm.

To study subcellular localization of the peptides we used CKGC and SKGS peptides with N-terminally conjugated FITC. We first turned to HeLa cells that are convenient model for microscopic studies. The cells were incubated for 1 h with 100 μM of FITC-CKGC or FITC-SKGS peptides pretreated with DTT, then fixed and examined. Confocal microscopy (**Figure 6**) indicates punctate cytoplasmic localization of the fluorescence for both peptides, suggesting vesicular localization. The peptide-containing vesicles were found throughout the cytoplasm, but never in the nuclei. In the cytoplasm, the vesicles were mainly concentrated at the vicinity of cell nuclei, suggesting localization in the Golgi. To label the Golgi we turned to mCherry-TGNP-N-10 plasmid encoding for mCherry-tagged TGOLN2, a marker of trans-Golgi network. HeLa cells were transfected with mCherry-TGOLN2 and treated with FITC-conjugated CKGC or SKGS peptides. Confocal microscopy reveals that the majority of both peptides indeed co-localizes with the Golgi marker (**Figure 6**). To find out if the peptides also reside in the mitochondria, HeLa cells were transfected with pDsRed2-Mito plasmid encoding DsRed fused to mitochondrial targeting sequence of cytochrome c oxidase. As shown in **Figure 6**, CKGC was not found in the mitochondria. Both FITC-CKGC and FITC-SKGS were also internalized by Jurkat cells (**Supplementary Figure 2**). Co-localization of the peptides with Golgi markers was not possible to study in these cells, as the fluorescence of FITC was rapidly quenched by detergents during immunocytochemistry, and the cells were resistant to transfection. However, the localization pattern of the peptides is similar to that in the HeLa cells. Both peptides were mainly found in the vesicles that located mostly at the groove of the nucleus. Such grooves were also labeled by antibodies to GM130, a marker of cis-Golgi compartment (Matlik et al., 2015) in the separately stained Jurkat cells (**Supplementary Figure 2A**). Thus, although not revealed with certainty, the peptides seem to localize in the Golgi also in the Jurkat cells. GM130 antibody also stained juxtanuclear regions in the HeLa cells, reminiscent of the localization of the FITC-CKGC peptide (**Supplementary Figure 2B**).

### CKGC Peptide Promotes Survival of Embryonic Dopaminergic Neurons

**FIGURE 7:**
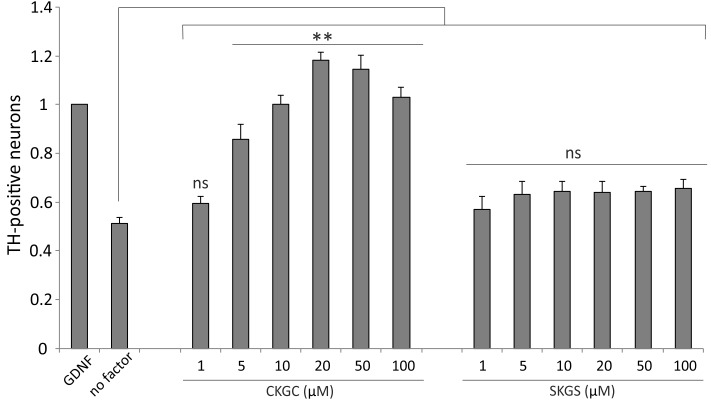
CKGC peptide promotes survival of cultured embryonic dopaminergic neurons. Dissociated cells of the embryonic mouse midbrains were cultured in the presence of CKGC or control SKGS peptides at indicated concentrations for 5 days. The dopaminergic neurons, recognized by antibodies to their specific marker tyrosine hydroxylase were counted under the microscope and expressed as percentage of the neurons grown in the presence of survival-promoting factor GDNF. Shown are the means ± SEM, *n* = 7 (independent cultures). Statistical significance between the groups was calculated by one-way ANOVA with Dunnett’s post-test comparing the experimental groups to the group grown without any added factor (indicated by long vertical line). ^∗∗^*p* < 0.01; ns, not significant.

Finally, we studied whether the CKGC peptide could promote survival of primary dopaminergic neurons *in vitro*. We cultured the cells from dissociated embryonic mouse midbrains, containing the dopaminergic neurons among other neurons, in the presence of increasing concentrations of CKGC peptide or the control SKGS peptide for 5 days. The number of dopaminergic neurons, revealed by tyrosine hydroxylase immunostaining, was then microscopically determined. As the natural survival-promoting factors were lost in the culture conditions, the dopaminergic neurons gradually died (**Figure 7**, no factor group), whereas GDNF, known to be survival-promoting for these neurons (Yu et al., 2008), prevented this death. The CKGC peptide dose-dependently antagonized the death of dopaminergic neurons, being at 20–50 μM concentrations even more effective than GDNF, used for normalization of the data (**Figure 7**). The control SKGS peptide had only slight survival-promoting effect that did not reach statistical significance (**Figure 7**).

## Discussion

Mesencephalic astrocyte-derived neurotrophic factor and CDNF are proteins that potently protect the cells against death, both *in vitro* and *in vivo* (Voutilainen et al., 2015; Lindholm et al., 2016; Lindahl et al., 2017). Although the survival-promoting mechanism of MANF is not clear, it critically depends on the CXXC motif, as destruction of the motif by mutation of Cys151, one of its cysteines, leads to complete inactivation of MANF (Matlik et al., 2015). The CXXC motif is located in the unstructured loop of the C-terminal domain of MANF and, in such exposed position, could directly participate in the survival-promoting activity of MANF, although its mode of action is currently unknown. We realized that this motif could have cytoprotective ability on its own as a tetrapeptide. In this study, we set up to characterize the survival-promoting properties of the CKGC peptide on cultured cells. The main results of this study are: (i) CKGC peptide weakly protects the Jurkat cells against three death programs: necroptosis, mitochondrial apoptosis and death receptor-mediated apoptosis, the latter program being antagonized most potently. (ii) Reduction of the cysteines with DTT potently improves cytoprotective potency of the peptide, most prominently against death receptor-activated apoptosis. (iii) Order and identity of amino acids in the peptide are essential for its activity. (iv) The peptide reduces ROS levels, maintains mitochondrial membrane potential and prevents caspase activation via its cysteine residues. (v) The peptide enters the cells and has vesicular localization in the cytoplasm and Golgi complex. (vi) The peptide potently protects cultured dopaminergic neurons against programmed cell death.

To study the cytoprotective mechanism of the peptide, we turned to Jurkat cells as a model, as these cells can be induced to die via several death modes (Valavanis et al., 2001; Karpinich et al., 2006; Li and Shively, 2013; Sosna et al., 2014). The peptide potently protected the cells against death receptor-induced apoptosis whereas its effects against other tested cell death programs (mitochondrial apoptosis and necroptosis) were only modest. Fas receptors have important role in the physiology of T cells, allowing selective elimination of the infected or cancer cells (Strasser et al., 2009). The functional importance of Fas pathway in Jurkat cells may explain why the effect of CKGC was most efficient against Fas-mediated apoptosis. We decided to restrict our further studies on the cytoprotective mechanism of the peptide to the activated Fas-induced model. However, the broad range of survival-promoting activity of the peptide should be kept in mind.

We present evidence that CKGC peptide protects Jurkat cells against activated Fas-induced apoptosis via its cysteine residues. (i) The control peptide where the cysteines were replaced with serines was not able to protect the cells. Taking into account a similar charge and spatial arrangement of the cysteine or serine residues in the CKGC and SKGS peptides, respectively, we can assume that the thiol groups of the cysteines must be responsible for the cytoprotective effect. (ii) Reduction of the cysteines by DTT drastically improved the anti-apoptotic properties of the peptide, obviously via keeping the highly reactive thiol groups of CKGC in reduced state and thereby preventing polymerization of the peptides. As expected, the DTT treatment did not improve the cytoprotective properties of the control SKGS peptide. (iii) The dCKGC, composed of amino acids in D-conformation was fully active, suggesting that it does not protect the cells via interacting with other proteins (at least as a main mechanism), as those interactions are most probably lost in D-enantiomer, whereas the thiol groups remain active in both D- and L-enantiomers (Ferrari et al., 1995).

We show that the position of cysteines in the sequence of the peptide, as well as the identity of other amino acids are also important for the functionality of the peptide. In particular, lysine could be essential, as due to its size and positive charge it could have a stereochemical impact on the other amino acids in the chain. Actually, the CXXC motif of human MANF (CKGC), human CDNF (CRAC), and MANF of the fruit fly (CDGC) all have a polar amino acid with a long side chain (Lys, Arg, and Asp) on the second position and a small neutral amino acid (Gly or Ala) on the third (Palgi et al., 2009). This rule may be important for the proper conformation of the motif. We compared cytoprotective ability of the peptides with different distance between two cysteines and different location of the lysine: GCCK, GCCG, KCCG, CGCK, and the original CKGC peptide, and got the following row of efficiency: GCCK > GCCG = KCCG = CKGC > CGCK. An obvious difference between the GCCK, GCCG, and KCCG peptides is the position of lysine, so we assume that the side chain of this amino acid affects the activity of tetrapeptide depending on its position, the most efficient position being at the C-terminus. Concerning the amino acid chain length between two cysteines, the efficiency of the peptides lowers as follows: CXXC = XCCX > CXCX and could likely be explained by the stability of disulfide bond, formation of which is preferable in the peptides with an even number of amino acids between two cysteines. Herewith, the absence of intervening amino acids is also more preferable than the presence of one or three (Zhang and Snyder, 1989). This may explain the lower efficiency of CGCK, which has one amino acid between the cysteines in comparison with other tested peptides having two or zero intervening amino acids. Thus, the cytoprotective activity of CXXC peptide is influenced by the type and number of amino acids intervening and flanking the two cysteines. However, all tested peptide variants were functional and thus the main cytoprotective function is based on the reaction of cysteines. This conclusion is also supported by the fact that the efficiency of all these cysteine-containing tetrapeptides was proportionally improved by preincubation with DTT (not shown). Also, CKGC peptide that consists of dCKGC did not lose its cytoprotective ability, which would probably happen if the spatial structure of the peptide would be critically important. Of note, the dCKGC protected the cells and inhibited caspases even more efficiently than the original L-isomeric form of the peptide. The reason for this is unclear, but possibly the peptide composed of dCKGC, that are uncommon in the mammalian cells, is more resistant to proteases (Shi et al., 2014).

A pair of reduced thiols is a common antioxidative mechanism in the cells and is found in glutathione, thioredoxin, oxidoreductase, and other systems (Wouters et al., 2010; McBean et al., 2015). In particular, neutralization of excessive ROS, generated during oxidative stress in the cells is carried out via CXXC motifs of these proteins. Uncontrolled ROS generation is a part of cellular damage mechanisms in the apoptosis, and is involved in many degenerative diseases (Bhat et al., 2015; Angelova and Abramov, 2018; Hoffmann and Griffiths, 2018). Although less studied than in the mitochondrial apoptosis, ROS are clearly involved in the death receptor-mediated apoptosis (Gulbins et al., 1996; Vercammen et al., 1998; Sato et al., 2004; Circu and Aw, 2010; Roberge et al., 2014), as also shown in this study. Indeed, we show that activated Fas increased the levels of ROS in Jurkat cells, and the CKGC peptide significantly counteracts this rise. The naturally high endogenous ROS levels in Jurkat cells were also lowered by the peptide. We believe that such antioxidant activity is part of the cytoprotective mechanism of CKGC peptide. Elimination of excessive ROS could participate in the prevention of caspase activation and maintenance the integrity of mitochondria.

In death receptor-mediated apoptosis, the initiator caspase-8 is activated in the death-inducing signaling complex at the cytoplasmic domain of the ligated death receptors. Active caspase-8 in turn cleaves and thereby activates the effector caspases-3 and -7 (Ashkenazi and Salvesen, 2014). We show that activation of both caspase-8 and caspases 3/7 by Fas-activating antibody is impeded by the CKGC peptide. ROS can inactivate anti-apoptotic Bcl-2 and prevent procaspase-3 processing (Liu et al., 2013; Martinez-Reyes and Cuezva, 2014). Also, excessive ROS leads to down-regulation of c-FLIP, the inhibitor of caspase-8 (Circu and Aw, 2010; Wilkie-Grantham et al., 2013). ROS can mediate mitochondrial and endoplasmic reticulum dysfunction in the death receptor-mediated apoptosis, thus their neutralization could reduce cell death (Inoue and Suzuki-Karasaki, 2013). However, we cannot claim that reduction of ROS is a sole caspase-blocking mechanism of the peptide, as ROS are probably not the main cytotoxic mechanism in death receptor pathway. The CXXC motifs can also regulate cellular processes via other activities, such as metal binding, as they are able to chelate zinc and copper (Collet et al., 2003; Wouters et al., 2010; Safaei et al., 2012), or alleviating the unfolded protein response (Woycechowsky and Raines, 2003; Baratz-Goldstein et al., 2016). The cytoprotective activity of CKGC peptide composed of dCKGC suggests that caspase inactivation could not occur via direct interaction with the caspases (or other upstream proteins). However, how exactly is caspase activation interfered by the CKGC peptide needs further studies.

Jurkat cells are so-called type II cells where apoptotic signaling, triggered by death receptor ligation at the plasma membrane is amplified at the mitochondria (Tafani et al., 2006; Inoue and Suzuki-Karasaki, 2013). Indeed, mitochondrial membrane potential was lost in Jurkat cells by activated Fas and this loss was prevented by the CKGC peptide. CKGC unlikely influences mitochondria directly, because peptide was not located in these organelles. Probably the mitochondrial damage is prevented by neutralization of ROS, as excessive ROS promote the imbalance of mitochondrial membrane potential (Zorov et al., 2014). The exact mechanism of mitochondrial maintenance remains to be studied.

Our microscopic analysis suggests that the CKGC (and also SKGS) peptide enters the cells mainly via endocytosis. Indeed, it localizes to the intracellular vesicles rather than diffusely in the cytosol. We cannot, however, exclude that some portion of the peptide also passes directly the plasma membrane, although it does not conform the rules for classical cell-penetrating peptides (Copolovici et al., 2014; Keaney and Campbell, 2015). Co-labeling of the cells with FITC-conjugated peptide and the antibodies to endosomal markers failed technically, therefore the exact identity of the peptide-containing vesicles remains currently unclear. Classically, the endocytosed vesicles are fused to lysosomes to degrade their content. We cannot exclude that part of the peptide in our experiments is also targeted to lysosomes and degraded, as the fluorescence of FITC is lost at low pH and thus cannot be detected in lysosomes. However, majority of the peptide was localized to the Golgi and must have therefore been following alternative intracellular routes (Jovic et al., 2010; Wang and Hung, 2012). Why is the peptide targeted to Golgi so massively is currently not clear. However, it is tempting to speculate that the cytoprotective function of the peptide is, at least partially, executed in the Golgi.

CKGC motif is essential for the survival-promoting function of MANF protein, as its mutation (CKGS) inactivates the whole protein, both intracellularly *in vitro* and extracellularly *in vivo* (Matlik et al., 2015). Corresponding CXXC motif is also found in the related protein CDNF. Although it is not yet known how this motif in MANF protein promotes cell survival, the mechanism could be similar to that reported here for the tetrapeptide. Indeed, we show that the CKGC peptide protects cultured dopaminergic neurons against programmed cell death. Namely these neurons degenerate in the Parkinson’s disease and are rescued by MANF and CDNF in the animal experiments (Lindholm et al., 2007; Airavaara et al., 2009; Voutilainen et al., 2009, 2011; Huotarinen et al., 2018). The cytoprotective effect of the peptide on the dopaminergic neurons was potent, showing that these neurons are sensitive to the protective mechanisms provided by the peptide. Thus, the MANF-derived cytoprotective motif could potentially be used as MANF mimetics. Supporting for this notion is the fact that the endocytotic vesicles observed in this study for the peptide *in vitro* are also reported for the full-length MANF protein in the striatal cells and dopaminergic neurons *in vivo* (Matlik et al., 2017). Small-molecule mimetics of proteins in the form of peptides are not a new approach. Several short peptides have been developed from the sequence of neurotrophic factors to facilitate the treatment of neurodegenerative diseases (Kazim and Iqbal, 2016). CKGC peptide could potentially be used in a similar way.

## Author Contributions

VB designed and performed the experiments with Jurkat cells, interpreted the data, and wrote the manuscript. L-yY performed the experiments with dopaminergic neurons. JP contributed to the experiments and data interpretations. UA provided intellectual input, direction in experimental design, provided resources, and edited the manuscript.

## Conflict of Interest Statement

The authors declare that the research was conducted in the absence of any commercial or financial relationships that could be construed as a potential conflict of interest.

## Abbreviations

MANF: mesencephalic astrocyte-derived neurotrophic factor
CDNF: cerebral dopamine neurotrophic factor
GDNF: glial cell line-derived neurotrophic factor
ROS: reactive oxygen species
FITC: fluorescein isothiocyanate
FBS: fetal bovine serum
DTT: dithiothreitol
PFA: paraformaldehyde
DAPI: 4′,6-diamidino-2-phenylindole
Nec-1: necrostatin-1
