# Supplementary material for: Antioxidative CXXC Peptide Motif From Mesencephalic Astrocyte-Derived Neurotrophic Factor Antagonizes Programmed Cell Death
Source: Front Cell Dev Biol. 2018 Sep 4;6:106. doi: 10.3389/fcell.2018.00106 (PMC6132022; doi:10.3389/fcell.2018.00106)
Supplement: Supplementary file 1 [file Data_Sheet_1.docx]

Supplementary Material

Antioxidative CXXC peptide motif from Mesencephalic Astrocyte-derived Neurotrophic Factor antagonizes Fas-mediated apoptosis

Valentina Božok, Jaan Palgi, Urmas Arumäe*

*** Correspondence:** Urmas Arumäe, Division of Gene Technology, Department of Chemistry and Biotechnology, Tallinn University of Technology, Akadeemia tee 15, Tallinn 12618, Estonia. e-mail: urmas.arumae@ttu.ee

## Supplementary Figures


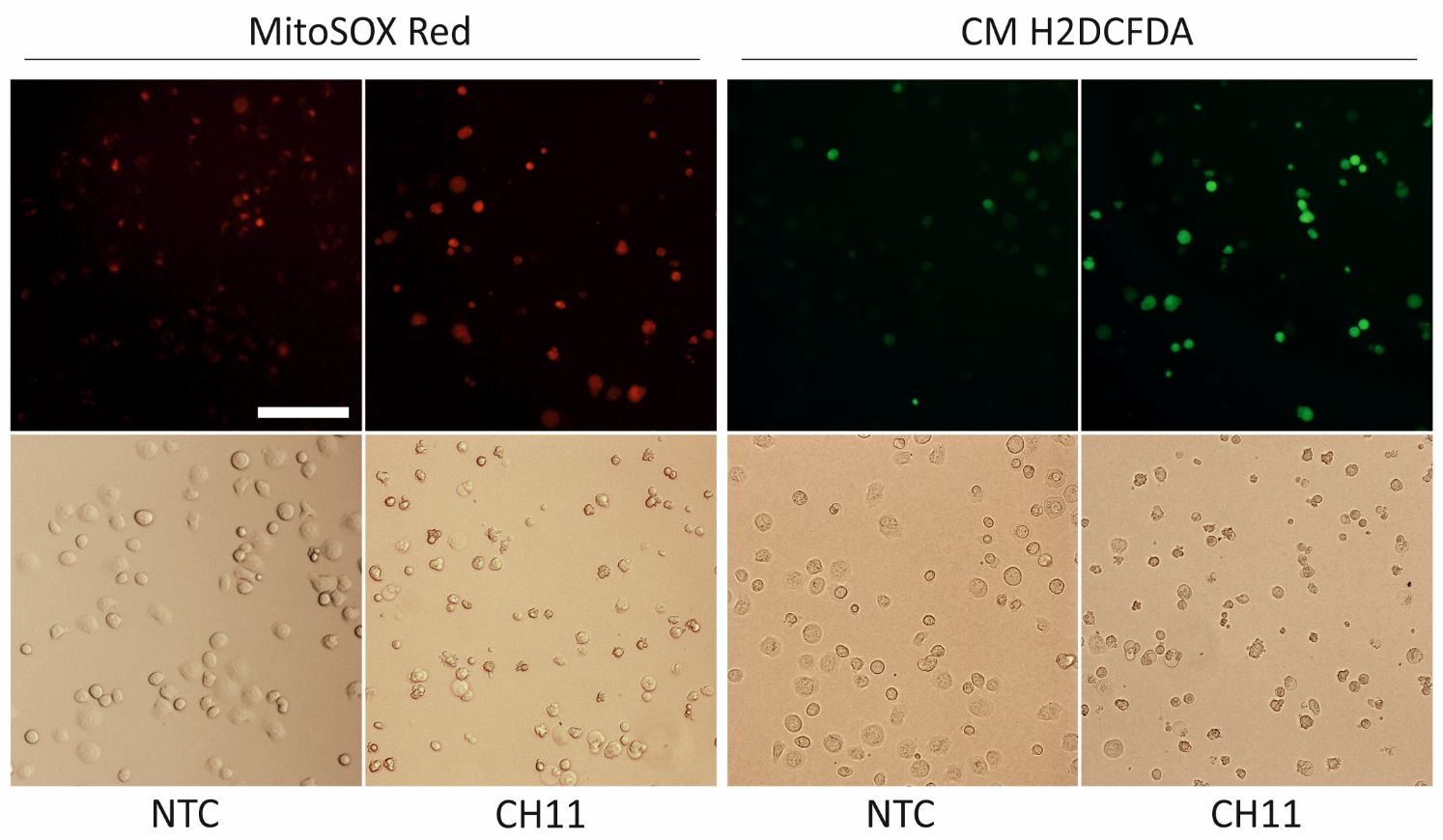


**Supplementary Figure 1.** **Activation of Fas receptor induces the rise of reactive oxygen species (ROS) levels.** Jurkat cells were incubated with anti-Fas agonistic antibody for 6 hours and stained with MitoSOX Red or CM-H2 DCFDA indicators of intracellular ROS. Upper panel shows the fluorescent images, the lower panel - the corresponding phase contrast images. Scale bar 100 μm.


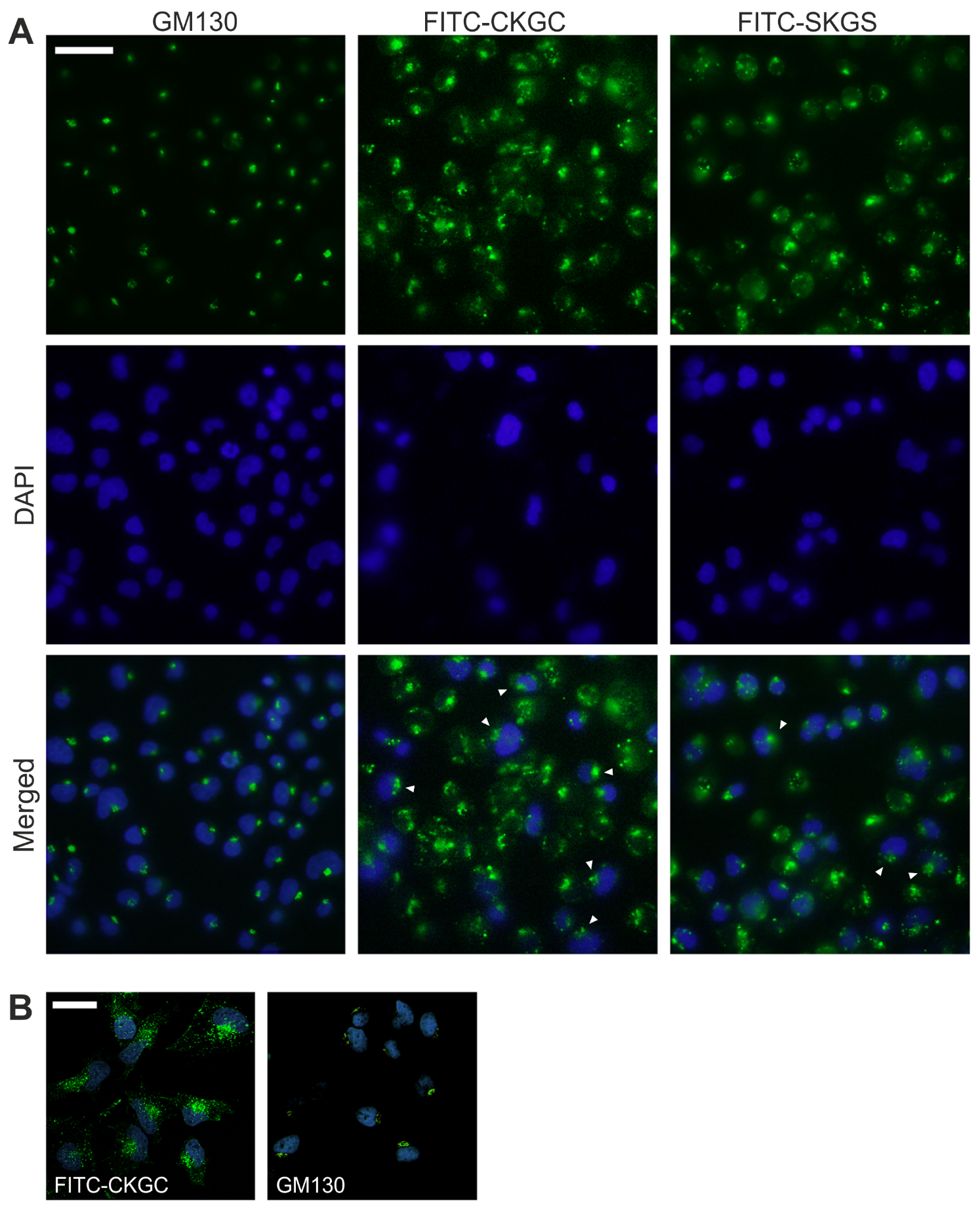


**Supplementary figure 2. Intracellular localization of the peptides in Jurkat and HeLa cells.** **(A)** Jurkat cells were incubated with 100 μM of CKGC or SKGS peptides with N-terminally conjugated FITC for 1 hour, attached to the coverslips in serum free medium and fixed with 4% PFA. Separate cells were immunostained with antibody GM130, specific to cis-Golgi compartment. The nuclei were labeled with DAPI. The images were taken by epifluorescent microscopy. Both GM130 and FITC are shown in green, DAPI in blue. Note the GM130-positive dots in the grooves on the surfaces of the nuclei, showing the localization of cis-Golgi network in Jurkat cells. Arrowheads on the images of FITC-peptide-loaded cells point to similar punctate structures on the nuclear grooves, suggesting localization of the peptides in Golgi. DAPI has not entered all FITC-peptide-loaded cells, most probably because the cells were not permeabilized by detergents that would rapidly quench the fluorescence of FITC. Scale bar, 30 μm. **(B)** HeLa cells were incubated with 100 μM of FITC-CKGC or immunostained for GM130 antibody, and examined by confocal microscopy. Note the juxtanuclear localization of the peptide and the cis-Golgi immunostaining. Scale bar, 30 μm.
